# Cases of Pericarditis Masked by Delirium

**Published:** 1854-01

**Authors:** Austin Flint

**Affiliations:** Louisville


### BUFFALO MEDICAL JOURNAL

AND

### MONTHLY REVIEW.

**VOL. 9.** JANUARY, 1854. **NO. 8.**

### ORIGINAL COMMUNICATIONS.

### ART. I.— *Cases of Pericarditis masked by Delirium.*

By Austin Flint, M. D.

In a former volume of this Journal,* I reported a case of pleuro-pneumonitis complicated with pericarditis, unaccompanied by a peculiar form of delirium, the local affections, so far as concerns the rational symptoms, being remarkably latent. The fact that pericarditis is occasionally so masked by cerebral symptoms that the latter (without knowledge of this fact) are likely to engross the attention of the practitioner, leading him to regard the affection as seated within the head, has but recently been pointed out. Dr. Watson, in his printed lectures on practical medicine, speaks impressively of this fact. But in the late work of Dr. George Burrows, on disorders of the cerebral circulation, is a fuller consideration of the subject. Dr. B. gives an account of its literature, adducing a number of cases which have been communicated, within a few years past, by different writers. In connection with the report for this Journal referred to, the reader will find extracts from that portion of Dr. Burrows' work which pertains to the subject.

* No. for April, 1850. Vol. V., page 505.

Since that report was made, two cases illustrating the coincidence of delirium and pericarditis have fallen under my observation.

One of these cases was observed at the Buffalo Hospital of the Sisters of Charity in 1851. The notes of this case, at the time I am writing, are not available, and an account of it must therefore be given from memory. It will not be difficult to do this sufficiently for all practical purposes, as the patient was seen by me but once. At my morning visit at the hospital, I found that, on the evening previous, a patient had been admitted greatly prostrated, and in a state of delirium. Nothing was obtained relative to the previous history of the case. The patient had not spoken since his admission. He lay with his eyes open, fixed, most of the time, in one direction, taking no notice of things or persons around him, making no reply to questions. A peculiarity in this case was, that the patient frequently ejected saliva, with force, and without any regard to its destination. His bed, and the floor in proximity thereto, was covered with spittle. Persons in proximity to him were in danger of receiving it on their persons, not from design, but because it w[illegible] scattered at random, the patient not changing his position, but lying constantly on the back. Under these circumstances I deferred an examination of the case, and at my next visit I found the patient had died. The circumstances pertaining to the mode of dying I am unable to state from recollection. At the time I was observing the patient, the idea of pericarditis did not occur to me, but in thinking of the case afterward, a resemblance in the character of the delirium to that in the case of Crotty, formerly reported by me, led me to suspect this disease; so that, before the autopsy was made, I ventured to predict that it would be discovered. My predictions proved to be true. Recent pericarditis existed, and the heart was preserved as a specimen of that affection. With respect to the condition of other organs, I am unable to state without reference to the record made at the time.

Another case illustrating the occurrence of pericarditis masked by delirium has recently been under my observation at the Louisville Marine Hospital. In this instance, much to my surprise, considering the gravity of the symptoms, the patient emerged from the delirium, and, at the present moment, is convalescent, with, as I suppose, a heart permanently damaged. An account of this case I propose to give, with as much detail as shall seem advisable, taking the facts from the daily records at the bedside.

John Maher, aged 24, Irishman, laborer, admitted October 24, 1853. On attempting to obtain the previous history of the case, Dr. Dickinson, resident physician at the hospital, found him too dull to give any connected account either of past or present symptoms. So far as he could gather any information from his disconnected replies to questions, he thought his disease was intermittent fever, and directed twenty grains of the sulphate of quinia to be given in divided doses during the following twenty-four hours.

At 8 or 9 o'clock, P. M., he had chill and rigors, followed by febrile movement and sweating.

On the 25th he appeared delirious, frequently getting out of bed, and appearing to be bewildered. He talked but little. During the night of this day he got up several times without any apparent object, and was taken back to the bed by the ward attendant. Once, after getting out of bed, he fell to the floor apparently from weakness.

On the morning of the 26th he was found to be in a state of unconsciousness. On this morning, for the first time, my attention was directed to the case. The description recorded by me at the bedside was as follows:

The patient has lost one eye. The other eye remains open. He takes no observation of persons or things around him. The pupil is dilated. He winks frequently, and always when the finger is brought into close proximity to the eye. He maintains the dorsal position. Remains taciturn, and cannot be roused to reply to questions, or take notice of any thing. The skin is bathed in perspiration, sweat standing in drops on the face. Urine has been emitted freely in bed. The salivary fluid collects and escapes at the angles of the mouth. He does not swallow when drink is placed in the mouth. Respirations 36 per minute, and somewhat labored, but rhythm normal. Pulse 108, and moderately full.

The impulse of the heart extends over an area of from two to three inches in diameter; the lower boundary being about half an inch below the nipple. The nipple is nearly the center of the area of impulse. The impulse is forcible. There is dullness on percussion below the lowest point of impulse, and over an abnormal area in the precordia, the precise extent not determined. The heart sounds are unattended by *bruit* of any kind.

A blister, 6 × 6 was directed to be applied over the precordia.

The foregoing is all that was recorded at this period in the history of the case, relative to physical rigors. The examination was not so complete as, afterward, I could have desired. With reference to physical explorations, too, I had occasion, for some time afterward, to regret that the precordia had been vesicated, inasmuch as they were precluded by the condition of the surface. I may add that at this time, and for some time afterward, I supposed that the nature of the affection would soon be determined by an autopsy. The death of the patient was daily expected. To the reader practically acquainted with the subject of physical diagnosis in its relations to pericarditis, it is submitted now, for the physical signs already stated indicate the existence of that affection. The demonstrative physical proof of its existence, viz., a friction sound, was not discovered. The physical signs existing at a subsequent date will be presently stated.

*Oct.* 27. The patient remained through the afternoon of the 26th inst., in the same condition as that described in the morning record; but at 9 in the evening he had a partial return of consciousness. He was tolerably quiet during the night, occasionally changing his position. This morning he has been very restless, tossing about, and throwing himself from the bed, so that it became necessary to transfer him to a bed on the floor. He frequently groans and mutters. He has taken neither food nor drink since yesterday. Fluids introduced repeatedly into the mouth were neither swallowed, nor voluntarily ejected, but remained in the mouth until they run out. No dejection, but urine passed freely in bed. At the time of the daily record he lay with his eye open, frequently winking, the pupil dilated. The eye did not close on approximation of the finger till the eye lashes were touched. The respiration was labored, the expiration somewhat prolonged. The skin was cold. Pulse scarcely appreciable.

The blister on precordia produced good vesication.

*Oct.* 28. Patient was quiet during the night. He has taken no drink or nourishment since the 25th inst. He has lately resisted the introduction of fluids into the mouth by closing firmly the teeth. On making efforts to cause him to swallow, at the time of the examination, he resisted, but he swallowed a portion of the liquid introduced into the mouth. The skin on this morning was warm; the pulse more developed than on yesterday, and 80 per minute. Respirations 28, and somewhat labored; inspiration abnormally intense, with dilation of *alæ nasi.* Occasional moaning with expiration. No dejection. Has urinated freely in bed.

Spirit and water, and beef essence prescribed, to be given as freely as practicable.

*Oct.* 29. Marked improvement, the patient lying tranquil, eye opened, taking some notice of persons and things around him. Respiration normal. Skin warm and mellow. Pulse tolerably developed, soft, and not accelerated. During the latter part of preceding night he talked much, and loudly, frequently shouting. Took food this morning, viz., tea and bread. Had a dejection, and urinated freely — both in bed. Was not rational. Did not reply coherently to questions. This peculiarity was noticed, viz., he directed his vision to some point, now a portion of the pillow, and now his hand, protruded his tongue toward it, and then slowly grasped it with his lips and teeth. This he repeated several times during the examination.

The precordia was quite sore from the blister, preventing physical exploration. At the inferior portion of chest, posteriorly, the percussion sound was clear.

Treatment had consisted of diffusible stimulus, carbonate of ammonia, and the essence of beef. The spirit and ammonia were suspended on this day, and nutritious diet continued.

*Oct.* 30. Through the previous day and night, patient, most of the time, was restless, throwing himself about, getting up, calling names of different persons. On this morning his expression was idiotic. His eye was open, and he looked about with a vacant stare. He resisted, moderately, attempts at a physical exploration. Twice he said, while the record of symptoms was being made, "I beg pardon." These words were uttered spontaneously, with great slowness and hesitancy. He did not reply to questions, and was taciturn the greater part of the time. He did not utter any connected sentences. Protruded his tongue very slowly when requested. No dejection, but free urination in bed. Took food and drink when presented, eating very slowly and looking at the attendant with an expression devoid of intelligence.

The blistered surface in precordial region prevented a satisfactory physical exploration. The action of the heart, denoted by the sounds, was irregular. A point of impulse is neither seen nor felt, but, owing to soreness from the blister, palpation could be employed but imperfectly.

Clearness of resonance on percussion existed at the lower part of the chest, posteriorly, on both sides.

Marked tenderness on pressure (irrespective of the blister) existed at the lower part of the chest in front.

Skin cool. Pulse 48, and quite feeble. Respirations, 13.

Diffusible stimulus, in small quantities, was directed, with nutritious diet. No other treatment.

*Oct.* 31. Had an attack of convulsions in the night time, and another at 10, A. M. Pulse, 66. Respiration, 16. State of intellect not materially altered. Could not be made to protrude the tongue.

*Nov.* 1. Attacks of convulsions recurred three times during the preceding day, and once in the night. During these attacks the upper and lower extremities, and the muscles of the face, were convulsed with considerable violence. They were about an hour in duration. There was no foaming at the mouth, nor did the attendants notice any marked disturbance of the respiration. Urine passed in bed. No dejection. He frequently got out of bed, and appeared as if he fancied persons were in pursuit of him intending violence. He endeavored to break the walls of the room, and his hands were accordingly placed in a restraining apparatus. Protruded his tongue when requested, and kept it protruded till told to withdraw it.

Talked and shouted incoherent words much of the night.

Respirations not accelerated, and normal in rhythm. Pulse, 78 and feeble, Resisted efforts to examine the precordia.

*Nov.* 2. Restless night, rolling about the room, and shouting.

Copious dejection in bed. Asks for nothing, but takes food and drink readily when presented. Pulse, 90 and quite feeble. Skin cool. Respirations, 20.

*Nov.* 4. On the 2d instant he continued wakeful, shouting, with occasional manifestations of hilarity. But on this day he became quiet, and slept most of the time. Took food and drinks readily, and asked for bread. Did not reply to questions generally, but, in answer to how he felt, said he felt well. Protruded tongue after repeated directions to do so, as before, very slowly. Pulse, 108. Skin warm and moist.

The blistered surface was nearly well, but tenderness on pressure existed, not only over the sore surface, but in the neighborhood.

*Nov.* 6. Patient rational, replying to questions readily. Said he felt well, except that he had some pain in left breast above the nipple, sharp, and felt on taking a full inspiration.

*Nov.* 7. *Physical Signs.* Over precordia transversely through the line of nipple flatness on percussion as far as the nipple. Vertically dullness distinct but not marked over second rib, flatness from third rib.

Impulse extremely feeble, being just appreciable between fourth and fifth ribs, about half an inch below, and the same distance to the right of a vertical line passing through the nipple.

*Nov.* 8. Reported feeling quite well, without pain, but had had pain the previous night in left side over the third rib. Placed his hand in that situation when requested to indicate the seat of pain. Said that the pain was short, and particularly acute when he coughed. Had no recollection of the events of the previous fortnight. Stated that he was ill for two days before he came to the hospital, and that he suffered chiefly of pain in the left breast.

*Physical Signs.* Percussion-sound clear over both sides, exclusive of precordia; and the respiratory sounds on both sides healthy.

The heart impulse between the fourth and fifth ribs, half an inch below the level of the nipple, and the same distance to the right. Impulse at thi examination distinctly seen and felt. The point of impulse not materially changed by assuming the sitting posture. Action of heart quickened by this change of position. Notable dullness, amounting nearly to flatness, below third rib. Above third rib percussion-sound clear.

Flatness transversely from sternum of nipple, and dullness to the left of nipple. Breathing movements on both sides presented no disparity.

No bearing impulse in precordia. Sounds of heart normal.

Diarrhœa was somewhat troublesome at the date of this record.

*Nov.* 15. Improvement in intellect and strength had continued. He was now up and dressed, and able to walk about the ward.

*Physical Signs.* A movement seen in precordial region extending from the third to the fifth ribs. The visible movement not uniform at different points of the space over which it was apparent. There appeared to be an impulse between fourth and fifth ribs, and simultaneously, a visible depression (not impulse) between the third and fourth ribs, and just below the fifth rib. By palpation no impulse was appreciable except between fourth and fifth ribs, nearly on a level with the nipple, and half an inch to the right. In this situation the impulse was marked but not strong. Above, between third and fourth ribs, and below, between fifth and sixth ribs, there was no appreciable impulse, but evidently a *drawing in,* with the action of the heart, of the intercostal spaces.

On percussion, flatness existed transversely to about half an inch to right of nipple, three and a half inches from median line, and marked dullness to about an inch to the left of the nipple. Vertically, on line falling about an inch to the right of nipple, marked dullness over third rib, and flatness over fourth rib, and extending to lower part of chest. No friction, or any abnormal sounds discovered on auscultation.

At the time I am writing this report, Nov. 25, the patient is still in hospital. He complains of nothing but weakness. His appetite is good, and bowels regular.

I have given the history of this case, with a minuteness which may to some seem tedious, in order, *first,* that the reader may be able to judge of the correctness of the diagnosis. The interest and importance belonging to the case, depend, of course, on there being sufficient evidence of the existence of pericarditis. The points on which the diagnosis are based, to recapitulate briefly, are as follows: Increased area of dullness in precordia, together with abnormal degree of dullness, or flatness; elevation and diffusion of the impulse; irregularity and feebleness of heart's contractions, and, at the time of convalescence, in connection with persistence of the elevated impulse, depression in the intercostal spaces with the heart's action. I am free to admit that the results of more complete explorations during the progress of the affection would be desirable. But the condition of the precordia, and the state of the patient, rendered it difficult to make satisfactory examinations at that time. Farther efforts for this object, however, would have been made, had it seemed probable that the case would have terminated so favorably as it has done. In connection with the physical signs denoting pericarditis, it is to be considered that acute pulmonary disease was excluded by the absence of marked signs, as well as symptoms pertaining to the lungs. The symptoms (in distinction from signs) pointing to pericarditis were pain in precordia, which was the prominent symptom before the patient entered the hospital, and was experienced after his consciousness returned, together with tenderness in the same region evidently not dependent on the blister; and, during the severity of the disease, notable oppressions or embarrassment of the circulation.

Assuming the diagnosis to be correct, a *second* object of the report is to describe fully the cerebral symptoms, which were the prominent features in the case, and were well calculated to mask the cardiac affection. If the reader will take the trouble to refer to the case previously reported by me for this Journal, he will find a striking resemblance in the character of the delirium in that case, to that of the case now reported; and in both the distinctive traits are those observed in the cases referred to by Drs. Watson and Burrows. The peculiarities are so striking, that, after having observed a case, the practitioner is afterward led, at once, therefrom to recognize, the disease. The characters of the delirium are, stupor and wakefulness combined, the patient lying with the eyes opened, and fixed in one direction, not replying to questions, and insusceptible of being roused by any exertions for that end; this state followed by paroxysms of active delirium, the patient shouting, and apparently laboring under the fear of harm, with occasional manifestations of hilarity.

A striking point of resemblance in the present case, to that of Crotty, formerly reported, is, that each appeared to imagine he had committed some offence for which he was to be punished. In the case of Crotty, this was shown by his uttering, at one time, in reply to questions of any kind addressed to him, the word "guilty," and afterward asking why he was not hanged. In the case of Maher, among the first words spoken were, "I beg pardon," which were repeated several times, and nothing else said.

The delirium which characterized both cases was entirely different, so far as my observations go, from that belonging to other affections attended by mental aberrations. It was not like the delirium of delirium tremens, of continued fever, or of encephalitis; and, if the peculiarities which distinguished these two cases exemplify an uniform kind of delirium occurring in certain cases of acute pericarditis, its character is so *unique* and striking, that it should not only suggest the disease, but is entitled to be considered presumptive evidence of its existence.

In view of the consideration just stated, I trust that I shall not be thought to have devoted in this article too much space to the report of a single case, more especially when it is considered that similar cases are rarely met with.

LOUISVILLE, Nov. 27, 1852.

