# Editorial Department

**Published:** 1854-02

**Authors:** 


The following *errata* occurred in Dr. Flint's report of a case of pericarditis, in the January No. The correction arrived too late for that number, and is inserted in the present as the errors are important:

2d line, page 449, for "*unaccompanied*," read *accompanied.*

7th line from the bottom of page 451, for "*rigors*," read *signs.*

2d line, page 452, for the words "*now for*," read *how far.*

7th line, page 455, for "*bearing*," read *heaving.*

